# Prevalence and treatment of human epidermal growth factor receptor 2-altered non-small cell lung cancer: a retrospective analysis and systematic literature review

**DOI:** 10.3332/ecancer.2024.1734

**Published:** 2024-08-01

**Authors:** Ning Yi Yap, Komathi Perumal, Pathmanathan Rajadurai

**Affiliations:** 1 Laboratory, Subang Jaya Medical Centre, Subang Jaya 47500, Selangor, Malaysia; 2 Jeffrey Cheah School of Medicine & Health Sciences, Monash University, Bandar Sunway, Petaling Jaya 47500, Selangor, Malaysia; 3 ePink Health Sdn. Bhd., Shah Alam 40150, Selangor, Malaysia; 4 Faculty of Medicine, Universiti Malaya, Kuala Lumpur 50603, Malaysia

**Keywords:** HER2, Malaysia, next-generation sequencing, non-small cell lung cancer, Southeast Asia

## Abstract

Human epidermal growth factor receptor 2 (HER2) is known for its oncogenic activities in diverse cancers, including non-small cell lung cancer (NSCLC). However, the prevalence of *HER2* alterations in Malaysian NSCLC patients remains unreported. This study examined the prevalence and characteristics of *HER2* mutations and amplification in a Malaysian cohort. Additionally, a systematic review was conducted to evaluate the global prevalence of *HER2* alterations in NSCLC, as well as the efficacy of HER2-targeted therapies observed in clinical trials. NSCLC tumour samples received from October 2019 to December 2022 for next-generation sequencing diagnostics were included in the retrospective analysis. In this patient cohort, *HER2 *alteration was present in 5.8% of patients; 3.9% had *HER2 *mutations, 1.5% had *HER2 *amplifications and 0.4% were both *HER2*-mutated and amplified. *HER2 *exon 20 insertions were the most common *HER2 *variants, detected in 47/59 (79.7%) of *HER2*-mutated patients. Among cases with *HER2 *exon 20 insertions, the Y772_A775dup variant was found in 34 patient samples. *HER2*-mutated patients were significantly younger than non-*HER2*-mutants (61 versus 64 years old; *p* = 0.046) and were inclined to be female and never-smokers, albeit not statistically significant. Patients with *HER2* amplification were more likely to have progressed post-tyrosine kinase inhibitor therapy (*p* = 0.015). The systematic review highlighted a global variation in the prevalence of *HER2* alterations in NSCLC, ranging from 0.3% to 9.1% for mutations and 0.2% to 19% for amplification. Finally, phase II clinical trials involving *HER2-*altered NSCLC patients demonstrated promising treatment outcomes with trastuzumab deruxtecan, trastuzumab emtansine, pyrotinib, pyrotinib + apatinib and trastuzumab + pertuzumab + docetaxel. In conclusion, the prevalence of *HER2* alteration among Malaysian NSCLC patients falls within the global range. A systematic review of clinical trials revealed promising treatment outcomes and Malaysian NSCLC patients with* HER2* alterations are anticipated to similarly benefit from *HER2*-targeted therapies.

## Introduction

Human epidermal growth factor receptor 2 (HER2), also known as ErbB2, is a transmembrane glycoprotein receptor exhibiting intracellular tyrosine kinase activity [1]. It consists of extracellular, transmembrane and intracellular domains, and plays important roles in various cellular functions including adhesion, differentiation, growth, apoptosis and migration [2, 3]. HER2 has garnered considerable attention due to its role in tumorigenesis and potential as a therapeutic target [3, 4]. Its aberrations have been implicated in the development and progression of various cancers, including non-small cell lung cancer (NSCLC) [3].

Three types of *HER2* oncogene activating mechanisms have been described in cancers, and they include gene mutation, gene amplification and protein overexpression [3]. These mechanisms of *HER2* activation have significant implications on treatment strategies, and prognostic outcomes which may differ according to the cancer type. *HER2* amplification and overexpression are well-established predictive markers for response to HER2-targeted monoclonal antibodies such as trastuzumab, in patients with breast and gastric cancers [2, 5]. However, HER2 protein overexpression has not demonstrated reliability in identifying NSCLC patients who may benefit from HER2-targeted therapies [6, 7]. In contrast, *HER2* mutations have shown greater promise in selecting NSCLC patients who are likely to respond to HER2-targeted therapies [8].

Currently, standard chemotherapy or immunotherapy is administered to patients with *HER2*-mutant NSCLC, but their effectiveness as second- or later-line treatment is limited [9]. Nonselective tyrosine kinase inhibitors (TKIs) have shown limited benefit in NSCLC patients with *HER2* mutation, with objective response rates (ORRs) ranging from 0% to 19% [10]. Trastuzumab-based chemotherapy was not found to be superior to chemotherapy alone whereas selective HER2 TKIs (e.g., poziotinib and pyrotinib) showed better activity in pre-treated NSCLC patients with *HER2* mutation [10]. More favourable data were reported in phase II studies evaluating antibody-drug conjugates (ADC) ado-trastuzumab emtansine and trastuzumab deruxtecan in *HER2*-mutated NSCLC patients [11, 12]. These agents bring hope to the management of *HER2*-altered NSCLC.

Malaysia is a multi-ethnic Southeast Asian country, comprising an ethnic Malay majority, as well as significant Chinese, Indian and indigenous populations. Lung cancer survival is the worst among all cancers in Malaysia; the overall 5-year relative survival for lung, trachea and bronchus cancer among Malaysian patients was 11.0%, with a median survival time of 6.8 months [13]. The prevalence of *HER2* mutation and amplification among NSCLC patients is not well-reported in the Southeast Asia region, and has not been reported in Malaysia. Therefore, we have performed a retrospective study to elucidate the prevalence of *HER2* alterations and the characteristics of NSCLC patients with these alterations, based on diagnostic next-generation sequencing (NGS) performed at a tertiary private referral medical center in Malaysia. Additionally, a systematic literature review was conducted to offer a comprehensive overview of the existing evidence concerning the prevalence of *HER2* mutation/amplification in NSCLC, as well as the efficacy of HER2-targeted therapies observed in prospective clinical trials involving NSCLC patients with *HER2* alterations.

## Methods

### Determining prevalence of HER2 mutations and amplification among NSCLC patients in Malaysia.

#### Patient samples

Tumour samples from lung cancer patients from several medical centers in Malaysia were collected and sent to the Subang Jaya Medical Centre (SJMC) laboratory for NGS. We analyzed the NGS results of consecutive samples received from October 2019 to December 2022, to determine the prevalence of *HER2* mutations and amplification. Ethical committee approval for the analysis of the retrospective NGS data was granted by the SJMC ethics committee (Ref: 201907.3 and Ref: 202109.3).

#### NGS testing

DNA from samples received from October 2019 to 2020 was sequenced using the Ion AmpliSeq Colon and Lung Cancer Research Panel v2 (Thermo Fisher Scientific, Waltham, MA, USA), while ribonucleic acid (RNA) was sequenced using the Ion AmpliSeq RNA Fusion Lung Cancer Research Panel or the Oncomine Focus Assay (Thermo Fisher Scientific, Waltham, MA, USA). DNA and RNA from samples received from November 2020 to December 2022 were sequenced using the Oncomine Precision Assay GX (Thermo Fisher Scientific, Waltham, MA, USA). The NGS testing process followed the method previously described by Rajadurai *et al* [14]. *HER2* mutations and amplification were detected using the targeted NGS panels.

#### Statistical analysis and data visualization

Statistical analysis was performed using the IBM SPSS Statistics Version 22.0 (IBM Corporation, Armonk, NY, USA). Patients’ demographic and clinical characteristics were evaluated using Pearson’s chi-square test or Fisher’s test for categorical variables, while the Mann-Whitney test was employed for comparing the patients’ age. The *HER2* mutation lollipop diagram was generated using the MutationMapper visualization tools available at the cBioPortal site (https://www.cbioportal.org/) [15].

### Systematic review of HER2 mutations and amplification in NSCLC

The systematic literature review was conducted in accordance with the preferred reporting items for systematic reviews and meta-analyses (PRISMA) guidelines [16].

#### Search strategy

A systematic literature review was conducted to obtain (a) the prevalence of *HER2* mutation/amplification in NSCLC, and (b) the efficacy of *HER2*-targeted therapies in NSCLC patients with *HER2* alterations. A comprehensive database search was performed in PubMed and Web of Science to identify relevant studies published up to 31 December 2022. The Medical Subject Headings and text word search terms used were (‘*HER2’* or ‘*HER-2’* or ‘*ERBB2’* or ‘*ERBB-2’*) AND (‘lung cancer’ or ‘NSCLC’). The studies were screened by two reviewers (NYY and KP) based on the article titles, abstracts and contents. The general inclusion criteria used to evaluate records include articles or abstracts published in the English language, and studies in which NSCLC patients were included. Studies involving only *in vitro* or *in vivo* samples, and review-type articles were excluded. The article selection criteria based on the populations, interventions and comparators, outcomes and study design (PICOS) are shown in Table 1.

Specific inclusion and exclusion criteria were also defined according to the two subtopics of the systematic literature review, as follows: (a) For systematic review on the prevalence of *HER2* mutations and amplification, publications reporting the prevalence of *HER2* mutations and amplification from a cohort of ≥ 200 NSCLC cases were included. Analyses involving only epidermal growth factor receptor (*EGFR*), Kirsten rat sarcoma 2 viral oncogene homologue (*KRAS*), anaplastic lymphoma kinase (*ALK*) or ROS proto-oncogene 1 (*ROS1*) wild-type cases, analyses using liquid biopsy samples only and reports of HER2 protein overexpression were excluded. (b) For a systematic review of clinical trials evaluating the efficacy of treatment in NSCLC patients with *HER2* mutations or amplification, prospective studies with ≥10 NSCLC subjects that were published from 2018 to 2022 were included. Retrospective studies and case series were excluded.

#### Data extraction

Data were extracted from articles that met the defined inclusion and exclusion criteria by one independent reviewer, and verified by a second reviewer. Data extracted for two subtopics of the systematic literature review were as follows: (a) For systematic review on the prevalence of *HER2* mutations or amplification, data extracted were on the prevalence of *HER2* mutations and amplification. (b) For a systematic review on prospective clinical trials evaluating the efficacy of treatment in NSCLC patients with *HER2* mutations or amplification, data extracted were the ORR, disease control rate (DCR), median progression-free survival (PFS) and median overall survival (OS).

## Results

### Prevalence of HER2 mutations and amplification in Malaysian NSCLC patients

#### Patient cohort

The demographic features of 1,373 NSCLC patients whose tumour samples were analyzed at the SJMC laboratory are shown in Table 2. Approximately half of the patients were male (52.7%), and the median patient age was 64 years old (range 16–93 years old). Most of the patients were of Chinese descent (76%), and nearly half of the patients were never smokers (44.8%). Most tumours (84.6%) were adenocarcinomas, and 88.1% of patients had advanced stage NSCLC (stage III or IV disease). Most patients (77.7%) were TKI-naïve.

#### NGS and HER2 profile of NSCLC patient cohort

**Figure 1.**
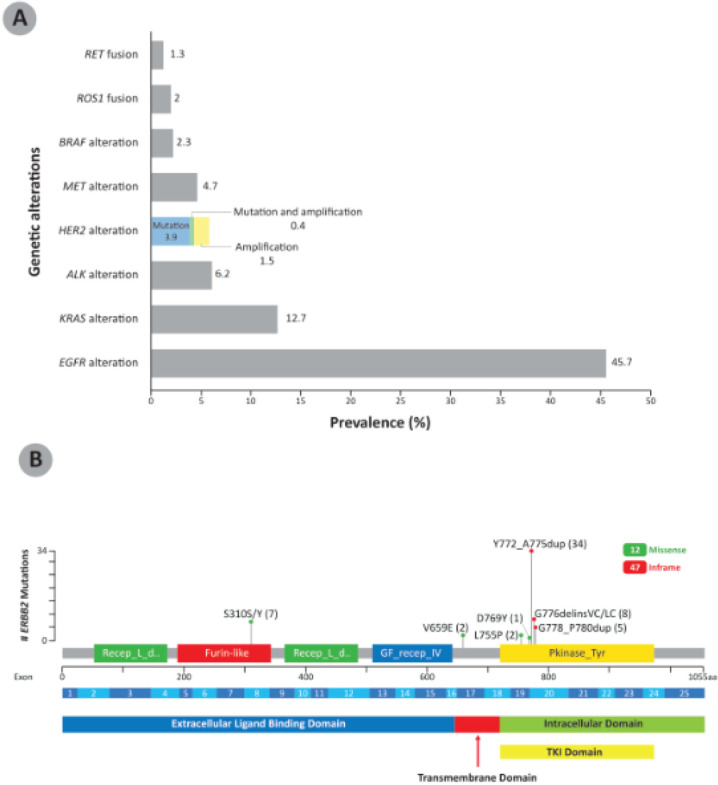
(a): Prevalence of genetic alterations in a cohort of NSCLC patients in Malaysia (N = 1,373). The breakdown of prevalence of HER2 mutation, amplification, as well as mutation and amplification are shown in color. (b): HER2 mutation variants reported in NSCLC patients in Malaysia (n = 59). The number of patients showing the specific mutations are indicated in brackets. HER2 exon 20 insertions were the most common HER2 variant in the NSCLC patients. BRAF: B-Raf proto-oncogene; MET: mesenchymal-epithelial transition; RET: RET proto-oncogene.

Among the NSCLC specimens analysed at our centre, nearly half (*n* = 627, 45.7%) showed *EGFR* alteration, followed by *KRAS* alteration (174, 12.7%) and *ALK* alteration (85, 6.2%). *HER2* alteration was present in 79 patients (5.8%); 54 (3.9%) of these were *HER2* mutations only, 20 (1.5%) were *HER2* amplification only and 5 (0.4%) were both *HER2*-mutated and amplified (Figure 1a). The *HER2* mutation variants reported in the NSCLC patients are shown in Figure 1b. *HER2* exon 20 insertions, found in the TKI domain, were the most common *HER2* variants among these NSCLC patients, with 47 out of 59 (79.7%) patients having this form of *HER2* alteration. The *HER2* exon 20 insertion Y772_A775dup was the most frequent *HER2* variant, found in 34 patient samples. Other *HER2* mutations are found in the extracellular ligand binding domain (S310S/Y, 7 patients) and transmembrane domain (V659E, 2 patients).

*HER2* exon 20 insertions were mutually exclusive with *ALK*, *BRAF*, *EGFR*, *RET*, *ROS1* and *MET* genetic alterations. Two patients with *HER2* Y772_A775dup harboured *KRAS* alterations, one with *KRAS* amplification and the other *KRAS* K117N. *EGFR* sensitising mutations were detected in four patients with *HER2* S310S/Y variants; of these, two patients were post-TKI progression cases. In addition, 11 patients with *HER2* amplification had *EGFR* sensitising mutation; of these, 5 patients were post-TKI progression. *TP53* mutations were the most common co-mutations seen with *HER2* mutation (14 patients) and *HER2* amplification (9 patients).

#### Characteristics of patients with HER2 mutations and amplification

Patients with *HER2* mutations were significantly younger than non-*HER2*-mutants (median age 61 versus 64 years old; *p* = 0.046), and were inclined to be female and never-smokers (not statistically significant; *p* = 0.111 and 0.204, respectively) (Table 3). On the other hand, patients with *HER2* amplification were inclined to be male, and ex- or current smokers (not statistically significant; *p* = 0.157 and *p* = 0.159, respectively). Patients with *HER2* amplification were more likely to have progressed post-TKI (*p* = 0.015). All five patients with *HER2* amplification who progressed post-TKI also had *EGFR* sensitising mutations.

### Systematic review of HER2 mutations and amplification in NSCLC

#### Study selection

**Figure 2.**
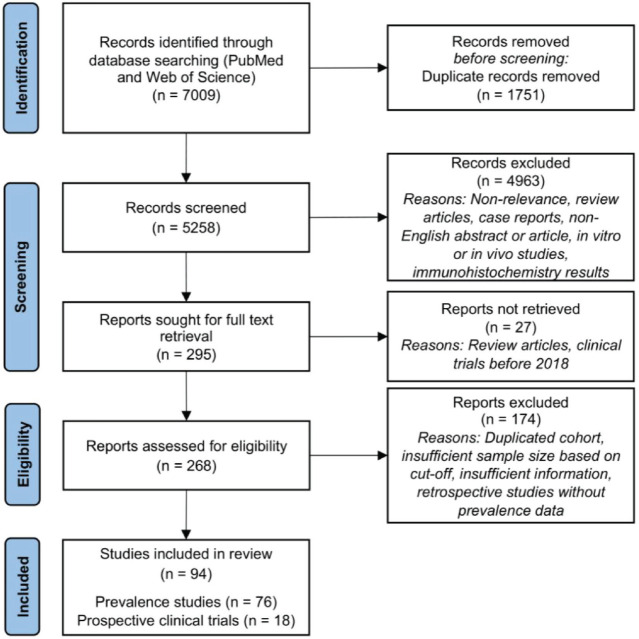
PRISMA diagram for inclusion of systematic review.

The database search performed on PubMed and Web of Science yielded 5,828 unique records for screening. Of these, 295 records were retrieved for full text screening; most records were excluded due to non-relevance, unsuitable article type (review articles and case reports), non-English abstract or article, unsuitable studies (*in vitro* or *in vivo* studies, and immunohistochemistry (IHC) results) or clinical trials performed before 2018. Upon applying the PICOS criteria, 94 articles were included; of these, 76 articles reported the prevalence of *HER2* mutation and/or amplification, and 18 articles were prospective clinical trials evaluating the efficacy of treatment in NSCLC patients with *HER2* mutations or amplification (Figure 2).

#### Prevalence of HER2 mutations and amplification

The prevalence of *HER2* mutations and amplification reported in global studies are shown in Table 4. *HER2* mutations in the studies were detected using various methods including Sanger sequencing, reverse transcription polymerase chain reaction (RT-PCR), NGS and matrix-assisted laser desorption ionisation-time of flight (MALDI-TOF) mass spectrometry (Supplementary Table S1 shows the full list of studies reporting the prevalence of *HER2* mutations and amplification). The prevalence of *HER2* mutations in NSCLC ranged from 0.3% to 9.1%. In some studies, the prevalence of *HER2* exon 20 insertions were specifically reported, ranging from 0.4% among African-American populations in North America, to 4% in North America and East Asia. *HER2* amplification was detected using fluorescent *in situ* hybridisation (FISH), silver *in situ* hybridisation (SISH), dual *in situ* hybridisation (DISH), multiplex ligation-dependent probe amplification (MLPA) or NGS. The prevalence of *HER2* amplification varied widely, from 0.2% reported in China, up to 19% reported in Japan [17, 18]. Two studies which reported relatively high prevalence of *HER2* amplification (14% and 19%) used the SISH or DISH method of detection [17, 19].

#### Efficacy of HER2-targeted therapies in NSCLC patients with HER2 mutations and amplification

Prospective clinical trials of various treatments for NSCLC patients with *HER2* mutations and amplification are shown in Table 5.

Two phase II trials of afatinib in NSCLC patients with *HER2* mutations were performed in post-progression patients; these trials revealed modest clinical benefits, i.e., ORR of 0%–7.7%, DCR of 53.9%–61.1%, median PFS of 2.8–4.0 months and median OS of 10–14 months. However, both studies did not compare the efficacy of afatinib in different *HER2* exon 20 insertion variants [20, 21].

Poziotinib achieved a higher ORR (27.8%–27.9%), DCR (70%–73%) and PFS (5.5 months) compared to afatinib in patients on subsequent lines of therapy [22, 23]. Poziotinib’s DCR and PFS at subsequent lines of therapy were comparable to its use in the first line setting (ORR of 41%, DCR of 73% and PFS of 5.6 months) [22–24]. However, poziotinib did not receive United States Food and Drug Administration approval due to its modest efficacy, yet significant gastrointestinal and dermal toxicities [25].

Treatment of lung cancer patients with trastuzumab emtansine at various lines of therapy yielded an overall ORR of 38.1%–51.0%, DCR of 52.4%–83.3% and PFS 2.8–5.0 months [8, 11, 26]. Li *et al* [8] reported comparable responses to trastuzumab emtansine among patients when stratified according to *HER2* status (mutation, amplification or combination of both). Although these trials recruited patients with central nervous system (CNS) metastasis, no subgroup analysis data were presented.

Pyrotinib has been investigated as a monotherapy, and in combination with apatinib for patients with *HER2* mutation or amplification [27–31]. The ORR, DCR and PFS were generally lower with pyrotinib monotherapy at 19.2%–30.0%, 74.4%–85.0% and 5.6–6.9 months, respectively, compared to 35.7%–51.5%, 93.9%–100% and 6.9–8.0 months seen in pyrotinib + apatinib [27–31]. In a subgroup analysis of pyrotinib monotherapy, the ORR were comparable between patients with and without brain metastases (25.0% versus 31.3%). 

Treatment of post-progression patients with trastuzumab deruxtecan (at a dose of either 5.4 or 6.4 mg/kg) in DESTINY-Lung01 and DESTINY-Lung02 yielded an encouraging ORR of 42.9%–54.9% and DCR of 90.4%–92.9% [12, 32]. In addition, trastuzumab deruxtecan at 6.4 mg/kg also yielded a more prolonged PFS of 8.2 months and OS of 18.6 months [12]. Trastuzumab deruxtecan seemed to achieve better treatment outcomes in post-progression lung cancer patients, compared to other *HER2-*targeted therapies (pyrotinib, afatinib, poziotinib and trastuzumab emtansine) (Table 5). In the DESTINY-Lung01 trial, comparable responses were observed with trastuzumab deruxtecan between patients with CNS metastasis and those without [12]. Safety-wise, although the DESTINY-Lung02 trial demonstrated similar efficacy of trastuzumab deruxtecan at both 5.4 and 6.4 mg/kg, a lower incidence of toxicities was observed with the 5.4 mg/kg dosage.

In the Drug Rediscovery Protocol (DRUP) trial, trastuzumab + pertuzumab demonstrated limited activity in patients with heavily pre-treated *HER2*-positive NSCLC (ORR 8.3%; DCR 38.0%; PFS 4.0 months; OS 10.0 months) [33]. Comparatively, trastuzumab + pertuzumab + docetaxel achieved improved outcomes in the IFCT 1703-R2D2 trial (ORR 29.0%; DCR 87.0%; PFS 4.0 months; OS 10.0 months) [34]. However, the IFCT 1703-R2D2 trial only included patients with stage III disease, while the DRUP trial recruited patients with metastatic disease.

Finally, neratinib as monotherapy as well as in combination with temsirolimus or trastuzumab in NSCLC patients with *HER2* alteration produced inferior ORR (0%–14%) and DCR (28%–49%) compared with poziotinib, pyrotinib, trastuzumab emtansine and trastuzumab deruxtecan.

## Discussion

This article aims to elucidate the prevalence of *HER2* mutations and amplification in NSCLC, as well as the clinical characteristics and mutational profiles of patients with these alterations, based on retrospective analysis of diagnostic NGS performed at a referral center in Malaysia. To the best of our knowledge, this article reports the first known statistics on *HER2* alterations among lung cancer patients in Malaysia. We also performed a systematic literature review to summarise the available evidence on the prevalence of *HER2* alteration in NSCLC and the treatment outcomes in these patients.

It is important to note that the frequency of *HER2* alterations may vary depending on the detection modalities used, target region of test assay, tumour heterogeneity, NSCLC subtype and sample type. Our systematic review on the prevalence of *HER2* mutations and amplification in NSCLC was analysed from a total of 76 articles; most articles described studies originated from East Asia, North America or Europe, with variations in the testing method used. The prevalence of *HER2* mutations reported may be higher in studies using NGS for testing, as more variants can be detected using this modality. In contrast, Sanger sequencing demonstrates lower sensitivity compared to NGS or RT-PCR. The assessment of *HER2* amplifications can be carried out utilising techniques such as FISH, SISH, DISH or NGS but currently, there is no standardised criteria for determining *HER2* amplification in NSCLC [1]. Finally, HER2 expression can be evaluated using IHC. The current testing recommendation is to include *HER2* mutation testing upfront as part of broad molecular profiling for NSCLC patients with advanced or metastatic disease, in particular, if approved therapies are available [1, 35].

In our retrospective analysis,* HER2* alteration was seen in 5.8% of Malaysian NSCLC patients. Of these, 3.9% had *HER2* mutations only and 1.5% had *HER2* amplifications only, and a small subset (0.4%) of our patient cohort were both *HER2*-mutated and amplified. Our prevalence findings fall within the range reported in global studies (0.3%–9.1% for *HER2* mutation and 0.2%–19% for *HER2* amplification) (Table 4). Specifically, the prevalence of* HER2* mutations (4.3%) was within the range reported by studies from East Asia (Table 4), with marginally lower overall prevalence from North America and Europe. The prevalence in this study was also slightly above Singapore (3.1%), the other Southeast Asian country with available published data. *HER2* exon 20 insertions were the most common *HER2* variants in our patient cohort. Similarly, available literature reported that most *HER2* mutations (90%) occur in the form of *HER2* exon 20 insertions, with Y772_A775dup (also referred to as A775_G776insYVMA, E770_A771insAYVM or A771_M774dup in scientific literature) being the most common subtype [3, 36]. Furthermore, in our patient cohort, *HER2* exon 20 insertions were mostly mutually exclusive to other driver mutations, with only the S310S/Y mutation found in the extracellular ligand binding domain co-occurring with *EGFR* sensitising mutations. This finding is also mirrored in another retrospective study, which found only eight patients (out of 12946 NSCLC patients) who had both *EGFR* and *HER2* mutations; of these eight patients, six patients had sensitising *EGFR* mutations and exon eight *HER2* mutation (S310F/Y) [37]. However, it is unclear whether if a concurrent *HER2* S310X mutation will affect response to EGFR TKIs.

In our patient cohort, those with *HER2* mutations tend to be younger than non-*HER2*-mutants (median age 61 versus 64 years old; *p* = 0.046) and were inclined to be female and never-smokers (not statistically significant; *p* = 0.111 and 0.204, respectively) (Table 3). *HER2* mutations have been reported to be significantly associated with never-smokers, patients of Asian origin and female patients [38, 39]. There may be a higher prevalence of *HER2* mutations in NSCLC patients from East Asia, although this could be attributed to the greater number of studies conducted in this region. *HER2* amplifications, on the other hand, have also been described as a potential mechanism of acquired resistance to EGFR TKI, as FISH analysis has revealed that *HER2* was amplified in 12% of tumours with acquired resistance, versus only 1% of untreated lung adenocarcinomas [40]. Other gene amplifications (e.g., *EGFR* and *MET*) are also known to act as resistance drivers against targeted therapy [41, 42]. These gene amplifications may occur de novo, or develop post-progression. In our patient cohort, all five patients with *HER2* amplification who progressed post-TKI also had *EGFR* sensitising co-mutations. These patients likely developed *HER2* amplification as acquired resistance to EGFR TKI. For these patients, therapies that target both *EGFR* and *HER2* may confer clinical benefit [43]. Future studies of the Malaysian NSCLC patient cohort with *HER2* alterations could benefit from analysis of treatment modalities and their impact on survival outcomes.

Our analysis of 18 prospective phase II clinical trials of various treatments for NSCLC patients with *HER2* alterations revealed promising treatment outcomes with trastuzumab deruxtecan, trastuzumab emtansine, pyrotinib, pyrotinib + apatinib and trastuzumab + pertuzumab + docetaxel. Both* HER2* mutation and amplification in lung cancer may be indicators of benefit with *HER2*-targeted therapy. *HER2* mutations particularly in the extracellular domain or kinase domain, as well as amplification, lead to *HER2* hyperactivation of downstream signalling cascades such as the PI3K and MAPK pathways [8]. Emerging therapeutic agents such as ADCs work by the selective binding of the monoclonal antibody component to the receptor’s extracellular domain, and delivery of the cytotoxic payload to arrest malignant cell growth. Anti-HER2 ADCs have generally demonstrated clinical activity in lung cancers with *HER2*-activating mutations, irrespective of the level of protein expression [8].

A phase II trial investigating treatment with trastuzumab emtansine in NSCLC characterised by HER2 overexpression or mutation was stopped early due to limited efficacy [44]. The authors noted that IHC 3+ or IHC 2+/FISH-positive tumours showed limited response to the investigational agent in the study [44]. In the phase II DESTINY-Lung01 study, trastuzumab deruxtecan was also evaluated in HER2-overexpressed metastatic NSCLC (IHC 2+ and 3+) at two dose levels: 6.4 and 5.4 mg/kg. The ORR was 26.5% and 34.1%, DCR was 69.4% and 78.0%, PFS was 5.7 and 6.7 months and OS was 12.4 and 11.2 months at 6.4 and 5.4 mg/kg, respectively. Both trastuzumab deruxtecan doses showed consistent antitumor activity in heavily pre-treated patients with HER2-overexpressed NSCLC [45]. This is in contrast with trastuzumab emtansine, which demonstrated limited efficacy in HER2-overexpressed NSCLC; DCR were only 7% and 30% in IHC 2+ and 3+ cohorts, respectively [7, 44]. Trastuzumab deruxtecan has an 8:4 8:1 chemotherapy drug-to-antibody ratio, compared with trastuzumab emtansine’s 3.5:1 chemotherapy drug-to-antibody ratio, which may explain the improved efficacy of trastuzumab deruxtecan [46]. Additionally, the membrane permeability of the cytotoxic payload of trastuzumab deruxtecan contributes to the bystander effect of inducing apoptosis in neighbouring tumour cells [12, 46]. Nonetheless, this higher drug-to-antibody ratio also leads to increased toxicities associated with trastuzumab deruxtecan treatment, in particular interstitial lung disease. Likewise, the DESTINY-Breast03 trial demonstrated that trastuzumab deruxtecan conferred better clinical benefit compared to trastuzumab emtansine in HER2-positive breast cancer [5]. The incidence of interstitial lung disease was reported to be higher in breast cancer patients treated with trastuzumab deruxtecan (15%) compared with trastuzumab emtansine (3%), although no grade 4/5 event was seen with either treatment [5].

The common *HER2* mutation, HER2 Y772_A775dup / A775_G776insYVMA, was identified to confer increased resistance to afatinib and chemotherapy treatments in patients with NSCLC [47–49]. However, it is unclear if this resistance extends to treatment with other HER2 TKIs and ADCs. NSCLC patients with *HER2* mutations have a higher incidence of brain metastases compared with patients with *EGFR* or *KRAS* mutations [50]. Moreover, *HER2* exon 20 YVMA insertion is also associated with a higher lifetime incidence of brain metastasis in advanced NSCLC, compared to non-YVMA cases [51]. This higher propensity for brain metastasis might contribute to the challenges faced in achieving effective responses to afatinib and chemotherapy treatments due to poor penetration of the blood-brain barrier. In the phase II trials for NSCLC patients with *HER2* mutations, sub-group analyses revealed comparable outcomes in patients with CNS metastasis who were treated with trastuzumab deruxtecan or pyrotinib. This finding is encouraging as it indicates that these treatment approaches could be effective in managing patients with CNS metastasis.* TP53* is a common co-mutation that may also affect treatment efficacy. Co-mutations in the TP53 pathway have been shown to confer additional resistance to afatinib therapy in lung cancer [52]. In breast cancer, *TP53*-mutated patients tended to have a worse prognosis with anti-HER2 TKI treatment compared to TP53-wild-type patients [53]. Given the frequent occurrence of *TP53* co-mutations in NSCLC patients, further investigation is warranted to better understand its implications for *HER2*-targeted therapies.

## Conclusion

In conclusion, in this retrospective analysis of diagnostic NGS performed at a referral center in Malaysia, *HER2* alteration was present in 5.8% of Malaysian NSCLC patients. Of these, 3.9% had *HER2* mutation, 1.5% had *HER2* amplification and 0.4% had both *HER2* mutation and amplification. Most (79.7%) of Malaysian NSCLC patients with *HER2* mutation had *HER2* exon 20 insertions, with Y772_A775dup being the most frequent *HER2* mutation variant. These findings fall within the range reported in global studies; the prevalence of *HER2* mutations in NSCLC reported in global studies ranged from 0.3% to 9.1%, whereas the prevalence of *HER2* amplification ranged from 0.2% to 19%. A systematic review of prospective phase II clinical trials of various treatments for NSCLC patients with *HER2* alterations revealed promising treatment outcomes with trastuzumab deruxtecan, trastuzumab emtansine, pyrotinib, pyrotinib + apatinib and trastuzumab + pertuzumab + docetaxel. Malaysian NSCLC patients with* HER2* alteration are anticipated to similarly benefit from the abovementioned *HER2*-targeted therapies.

## Conflicts of interest

PR declares consultancies and receipt of speaker fees from AstraZeneca and Thermo Fisher, as well as research grants from AstraZeneca and Roche. NYY declares conference travel support from AstraZeneca. KP declares no conflict of interest regarding the publication of this article.

## Funding

This study received grant support from AstraZeneca.

## Author contributions

*Conceptualisation*, NYY and PR; *Supervision*, PR; *Funding Acquisition*, PR; *Data Curation*, NYY and KP; *Formal Analysis*, NYY; *Writing – Original Draft Preparation*, NYY and PR; *Writing – Review & Editing*, NYY, KP and PR.

## Data availability

Data supporting the findings of this study are available from the corresponding author upon request.

## Figures and Tables

**Table 1.** Criteria for considering studies for systematic review, based on the population, intervention, comparator and PICOS structure.

	Inclusion criteria	
	Prevalence of *HER2* mutation/amplification in NSCLC	Efficacy of *HER2*-targeted therapies in NSCLC
Population	Patients with NSCLC and *HER2* mutations or amplification	
Intervention/Comparators	Any	Chemotherapy, immunotherapy, TKI, monoclonal antibody therapy or ADC
Outcomes	Prevalence of *HER2* mutations or amplification	ORR, DCR, median PFS and median OS
Study designs	Studies involving cohort of ≥ 200 NSCLC cases	Prospective studies with ≥10 NSCLC subjects published from 2018 to 2022

DCR: disease control rate; HER2: human epidermal growth factor receptor 2; NSCLC: non-small cell lung cancer; ORR: objective response rate; OS: overall survival; PFS: progression-free survival; TKI: tyrosine kinase inhibitor

**Table 2.** Clinical characteristics of Malaysian NSCLC patients whose tumour samples were analysed using NGS from October 2019 to December 2022.

Characteristics	Sample size (*N* = 1,373)
Median age, years (range)	64 (16–93)
Gender, *n* (%) Male	723 (52.7)
Ethnicity, *n* (%) Malay Chinese Indians Other	191 (13.9) 1044 (76) 54 (3.9) 84 (6.1)
Smoking status, *n* (%) Never-smoker Smoker Ex-smoker Unknown	615 (44.8) 134 (9.8) 219 (16.0) 405 (29.5)
Histology, *n* (%) Adenocarcinoma Adenosquamous Squamous cell carcinoma Large cell carcinoma Poorly differentiated NSCLC NSCLC with neuroendocrine differentiation Other	1,161 (84.6) 29 (2.1) 121 (8.8) 8 (0.6) 42 (3.1) 5 (0.4) 7 (0.5)
Specimen site, *n* (%) Lung Bronchial Pleura Chest wall Lymph node Bone Liver Brain Other	922 (67.2) 56 (4.1) 115 (8.4) 12 (0.9) 101 (7.4) 68 (5.0) 29 (2.1) 21 (1.5) 49 (3.6)
Sample type, *n* (%) Biopsy Fine needle aspirate Fluid cytology Resection Unspecified	961 (70.0) 9 (0.7) 23 (1.7) 24 (1.7) 356 (25.9)
Stage, *n* (%) I II III IV Unspecified	39 (2.8) 38 (2.8) 119 (8.7) 1,090 (79.4) 87 (6.3)
Treatment status, *n* (%) TKI-naïve Post-TKI progression Unspecified	1,067 (77.7) 89 (6.5) 217 (15.8)

NGS: next-generation sequencing; NSCLC: non-small cell lung cancer; TKI: tyrosine kinase inhibitor

**Table 3.** Characteristics of Malaysian NSCLC patients with HER2 mutations and amplification compared with patients without the HER2 alterations.

Characteristic	HER2 mutations			HER2 amplification		
	Without *HER2* mutation (*n* = 1,314)	With *HER2* mutations (*n* = 59)	*p*-value	Without *HER2* amplification (*n* = 1,348)	With *HER2* amplification (*n* = 25)	*p*-value
Median age, years (range)	64 (16–93)	61 (32–80)	0.046	64 (16–93)	63 (38–85)	0.892
Gender, *n* (%) Male Female	698 (53.1) 616 (46.9)	25 (42.4) 34 (57.6)	0.111	706 (52.4) 642 (47.6)	17 (68) 8 (32)	0.157
Ethnicity, *n* (%) Malay Chinese Indian Other	184 (14) 998 (76) 50 (3.8) 82 (6.2)	7 (11.9) 46 (78) 4 (6.8) 2 (3.4)	0.525	188 (13.9) 1,024 (76) 53 (3.9) 83 (6.2)	3 (12) 20 (80) 1 (4) 1 (4)	0.959
Smoking status, *n* (%) Never-smoker Ex- or current smoker	582 (63.1) 341 (36.9)	33 (73.3) 12 (26.7)	0.204	609 (63.8) 345 (36.2)	6 (42.9) 8 (57.1)	0.159
Stage, *n* (%) I-II III-IV	72 (5.9) 1158 (94.1)	5 (4.2) 51 (91.1)	0.379	76 (6) 1,189 (94)	1 (4.8%) 20 (95.2)	1.000
Treatment status, *n* (%) TKI-naïve Post-TKI progression	1,017 (92.1) 87 (7.9)	50 (96.2) 2 (3.8)	0.424	1,052 (92.6) 84 (7.4)	15 (75) 5 (25)	0.015
Histology, *n* (%) Adenocarcinoma Adenosquamous SCC	-	58 (98.3) 1 (1.7) -	-	-	22 (88) 1 (4) 2 (8)	-


HER2: human epidermal growth factor receptor 2; SCC: squamous cell carcinoma; TKI: tyrosine kinase inhibitor

**Table 4.** Prevalence of *HER2* mutations and amplification in NSCLC reported in global studies.

Region	Countries	Prevalence of *HER2* mutations	No. of studies	Prevalence of *HER2* exon 20 insertions only	No. of studies	Prevalence of *HER2* amplification	No. of studies
East Asia	China and Taiwan	1.9%–8.6%	18	1.6%–2.6%	8	0.2%–2.8%	8
	China (SCC only)	0.3%–9.1%	3	-	-	0.8%	1
	Japan and Korea	1.8%–4.9%	8	1.7%–4%	5	2.1%–19%	6
South Asia	India	-	-	1.5%	1	-	-
Southeast Asia	Singapore	3.1%	1	2.7%	1	-	-
North America	USA and Canada	1%–3.4%	10	3%–4% (0.4% among African-American)	4	0.4%–3%	5
South America	Brazil	4.9%	1	0.8%	1	-	-
Europe	Belgium, Finland, France, Germany, Greece, Italy, Spain and Switzerland	1.2%–3%	8	0.8%–1.7%	6	0.7%–9%	6
Australia	Australia	1%	1	-	-	-	-
Russia	Russia	-	-	-	-	6%	1

HER2: human epidermal growth factor receptor 2; SCC: squamous cell carcinoma

**Table 5.** Summary of phase II clinical trials of *HER2*-targeted therapies for NSCLC patients with *HER2* mutations and amplification.

Treatment^†^	Line of therapy	Phase (Trial name)	Sample size	Treatment outcome				References
				ORR (%)	DCR (%)	Median PFS (months)	Median OS (months)	
Afatinib	Subsequent line (post-progression)	II (NICHE)	13	7.7	53.8	4.0	14	[20]
		II	18	0	61.1	2.8	10.0	[21]
Poziotinib	First line	II (ZENITH20-4)	70	41.0	73.0	5.6	NA	[23]
	Subsequent line (post-progression)	II (ZENITH20)	90	27.8	70.0	5.5	NA	[24]
	Subsequent line (post-progression)	II	30	27.9	73.0	5.5	15	[22]
Pyrotinib	Subsequent line (post-progression)	II	60	30.0	85.0	6.9	14.4	[31]
	First line and subsequent line	II	78	19.2	74.4	5.6	10.5	[27]
Pyrotinib for *HER2* amplification only	First line and subsequent line	II	27	22.2	81.5	6.3	12.5	[28]
Pyrotinib + apatinib for *HER2* mutation and amplification	At least 2 prior lines	II (PATHER2)	33	51.5	93.9	6.9	14.8	[30]
Pyrotinib + apatinib	Subsequent line (post-progression)	II	14	35.7	100	8.0	12.9	[29]
Neratinib	NA	II (PUMA -NER-4201) & SUMMIT)	43	0–4.0	35.0–39.0	2.9–5.4	NA	[54]
Neratinib + temsirolimus or trastuzumab			95	8.0–14.0	28.0–49.0	4.0–4.1	NA	
Trastuzumab emtansine	Various lines	II	18	44.0	83.3	5.0	NA	[26]
	Subsequent line (post-progression)	II (JapicCTI-194620)	22	38.1	52.4	2.8	8.1	[11]
Trastuzumab emtansine for *HER2* mutation and amplification	Various lines	II	49	51.0	NA	5.0	NA	[8]
Trastuzumab deruxtecan (6.4 mg/kg)	Subsequent line (post-progression)	II (DESTINY-Lung01)	91	54.9	92.3	8.2	18.6	[12]
		II (DESTINY-Lung02)	28	42.9	92.9	NA	NA	[32]
Trastuzumab deruxtecan (5.4 mg/kg)			52	53.8	90.4			
Trastuzumab + pertuzumab	Subsequent line (post-progression)	II (DRUP)	24	8.3	38.0	4.0	10.0	[33]
Trastuzumab + pertuzumab + docetaxel	Subsequent line (post-progression)	II (IFCT 1703-R2D2)	45	29.0	87.0	6.8	17.6	[34]

† Studies recruited patients with *HER2* mutations unless stated otherwise (*HER2* amplification)

DCR: disease control rate; NA: not available; ORR: overall response rate; OS: overall survival; PFS: progression free survival

## Supplementary materials

Supplementary Table S1 provides the list of studies reporting the prevalence of *HER2* mutations and amplification.

**Supplementary Table S1.** Studies reporting the prevalence of *HER2* mutations and amplification.

Region	Histology type	Sample size	*HER2* mutations	*HER2* amplification	References
Prevalence	Detection method^†^	Prevalence	Detection method
Japan and Taiwan USA and Australia	NSCLC (Only found in adenocarcinoma)	269 and 145 157 and 100	3% and 1.4% (4% adenocarcinoma) 0 and 1% (0.4% adenocarcinoma)	PCR ex 18–24	-		[1]
China	NSCLC	49 adenocarcinoma 153 SCC 10 adenosquamous carcinoma	1 (2%) adenocarcinoma	PCR ex 19–20	-		[2]
China	Adenocarcinoma	981	27 (2.8%)	PCR ex 18–21	-		[3]
China	SCC	310	1 (0.3%)	PCR ex 18–21	-		[4]
China	Adenosquamous Adenocarcinoma	76 646	1.3% 2.6%	PCR ex 20 only	-		[5]
China	Adenocarcinoma SCC Adenosquamous LCC	1,356 310 57 19	2.5% 0.3% 1.8% 0	PCR All mutation	-		[6]
China	NSCLC	859	2.4%	PCR ex 20 ins and NGS	-		[7]
China	NSCLC	1,200	4.3%	NGS	2.4%	NGS	[8]
China	NSCLC	640	1.9%	RT-PCR	-		[9]
China	NSCLC	884	2.1%	NGS	2.1%	NGS	[10]
China	NSCLC	1,929	2.5%	N/A ex 18–21	0.2%	N/A	[11]
China	NSCLC	3,440	8.6%	NGS	0.9%	NGS	[12]
China	NSCLC	20,656	5.94% all mutations 1.4% ex 20 ins	N/A	-		[13]
China	SCC	488 (tissue and plasma)	9.06%	NGS	0.8%	NGS	[14]
China	NSCLC	1,087 tissue, 368 blood, 68 pleural effusion	5.6%	NGS	2.8%	NGS	[15]
China	NSCLC	21,745	3.1% activating mut	NGS	-		[16]
China	NSCLC	18,205	1.56% ex 20 ins	NGS ex 20 ins	-		[17]
China	Adenocarcinoma	8,247	2.5% ex 20 ins	NGS ex 20 ins	-		[18]
China	NSCLC	1,875	1.9% HER2 ins	PCR ex 20 ins	-		[19]
China	NSCLC	781	1.92%	RT-PCR or NGS All mut	-		[20]
China	NSCLC	1,497	2.9%	RT-PCR or NGS All mut	-		[21]
China	NSCLC	249	6.8%	N/A	1.6%	N/A	[22]
China	NSCLC	7,520	2.3% ex 20 ins	NGS ex 20 ins	-		[23]
China	NSCLC	1,270	1.7% ex 20 ins	NGS	-		[24]
Taiwan	Adenocarcinoma	888	4.5%	RT-PCR ex 18–21	-		[25]
Taiwan	NSCLC	1,001	1.7% HER2 mutation	MALDI-TOF	-		[26]
Japan	Adenocarcinoma	411	7 (1.7%)	PCR ex 20 ins	-		[27]
Japan	Adenocarcinoma	243	2.7% mut	PCR ex 20 ins	2.1%–3.7%	FISH or DISH	[28]
Japan	NSCLC	1,275 all 1,055 adenocarcinoma	3.6% all 4.3% adenocarcinoma	PCR ex 20 ins	19%	DISH	[29]
Japan	NSCLC	206	4.9%	NGS	-		
Japan	NSCLC	504	2.6%	PCR (ex 18–21)	-		[30]
Japan	NSCLC	3,441	4% ex 20 ins	NGS ex 20 ins	-		[31]
Japan	NSCLC (Only found in adenocarcinoma)	223	1.8% all, 2.6% adenocarcinoma	PCR ex 19–20	-		[32]
Japan	NSCLC	349	1.7% all	PCR ex 18–24	-		[33]
Japan	NSCLC	313	2.6% all 3.3% adenocarcinoma 1.3% SCC	PCR ex 19–20	-		[34]
Korea	NSCLC	271 adenocarcinoma	2.6%	NGS all mut	-		[35]
Korea	NSCLC	969	4.2%	NGS all	2.9%	SISH	[36]
Korea	NSCLC	1,108	2% ex 20 ins	NGS ex 20 ins	1.4%	NGS	[37]
Singapore	Adenocarcinoma	1,252	3.1% all 2.7% ex 20 ins	NGS	-		[38]
India	NSCLC	204	1.5%	PCR ex 20 ins	-		[39]
USA	NSCLC	344	4%	PCR ex 20 ins	-		[40]
USA	NSCLC	733 adenocarcinoma	3%	MALDI-TOF, PCR	-		[41]
USA (African American)	NSCLC	260	0.4%	NGS ins 20	-		[42]
China USA	NSCLC	490 2,200	3.5% 2.7%	NGS all	2.4% 1.5%	NGS	[43]
USA	NSCLC	1,674	3%	NGS	-		[44]
USA	NSCLC	6,832	3.4%	NGS all	3%	NGS	[45]
USA	NSCLC	1,006	1.3%	NGS	-		[46]
USA	Adenocarcinoma	920	3%	NGS (ex 20 ins)	-		[47]
USA	Adenocarcinoma	302	1%	NGS	-		[48]
USA	NSCLC	43,706	2.3% 1.5% Ex 20	NGS	2%		[49]
USA	NSCLC	570	5.1% ex 19 and 20	PCR, RT-PCR and NGS	0.4%	FISH	[50]
USA	NSCLC	12,946	1.5%	NGS all	1.1%	NGS	[51]
Canada	NSCLC	1,395	2.2% Ex 20 (1.6%) Transmembrane (0.3%)	NGS all	-		[52]
France	NSCLC	284	3%	N/A	-		[53]
France	NSCLC	11,723	0.8%	PCR ex 20	-		[54]
France	Adenocarcinoma	Caucasian 1,940 African 240 Asian 39	8.1% Asian 3.3% African 1.0% Caucasian	PCR ex 20 ins	-		[55]
France	Non-squamous NSCLC	2,921	2%	Not specified	-		[56]
France Switzerland Spain	Adenocarcinoma	3,800	1.7%	PCR (ex 20 ins)	-		[57]
Belgium	NSCLC	234	0.9%	NGS ex 20 ins	-		[58]
Finland	NSCLC	425	2%	NGS	-		[59]
Germany	NSCLC	398	1%	NGS ex 20	-		[60]
Germany	NSCLC	4,500	2.3% 1.5% ex 20 ins	NGS ex 20 ins and non-ex 20 ins	0.7% Five patients both mut and amp	NGS	[61]
Greece	NSCLC	502	1.8% all 2% adenocarcinoma	NGS all	1%	RT-PCR	[62]
Switzerland	Adenocarcinoma	469	1.9%	NGS all	-		[63]
Italy	Adenocarcinoma	537	3%	RT-PCR or NGS	1.9%	FISH	[64]
Netherlands	NSCLC	3,342	1.2%	NGS cohort ex 18–20	-		[65]
International cBioportal	NSCLC	1,934	4.3%	NGS all	-		[66]
Brazil	NSCLC	513	4.9% alterations	NGS all	-		[67]
Brazil	NSCLC	722	0.8%	NGS (ex 20 ins only)	-		[68]
Germany	NSCLC	378	-	-	2%	FISH	[69]
Germany	NSCLC	526	-	-	3.2% NSCLC, 6.3% in adenocarcinoma	FISH	[70]
France	NSCLC	250	-	-	9%	FISH	[71]
Japan	NSCLC	270	-	-	3% NSCLC	FISH	[72]
Korea	Adenocarcinoma	321	-	-	14.3%	SISH	[73]
China	NSCLC	7,643	-	-	2.8%	NGS	[74]
Russia	NSCLC	218	-	-	6% NSCLC	FISH	[75]
Poland	NSCLC	239	-	-	1%	MLPA	[76]

† Note: Detection method specified as PCR utilised PCR for amplification of nucleic acid and subsequent sequencing using conventional techniques, e.g. Sanger sequencing

amp: amplified; DISH: dual *in situ* hybridisation; ex: exon; FISH: fluorescent *in situ* hybridisation; HER2: human epidermal growth factor receptor 2; ins: insertion; LCC: large cell carcinoma; MALDI-TOF: matrix-assisted laser desorption ionisation-time of flight; MLPA: multiplex ligation-dependent probe amplification; mut: mutation; N/A: not available; NGS: next-generation sequencing; NSCLC: non-small cell lung cancer; PCR: polymerase chain reaction; RT-PCR: reverse transcription polymerase chain reaction; SCC: squamous cell carcinoma; SISH: silver *in situ* hybridisation; USA: United States of America
